# Addiction-like Eating in Chinese Adults: An Assessment Tool and Its Associations with Modern Eating-Related Habits

**DOI:** 10.3390/nu14224836

**Published:** 2022-11-15

**Authors:** Haofeng Ling, Juliet Honglei Chen, Kwok Kit Tong, Le Dang, Anise M. S. Wu

**Affiliations:** 1 Department of Psychology, Faculty of Social Sciences, University of Macau, Macao, China; 2 Centre for Cognitive and Brain Sciences, Institute of Collaborative Innovation, University of Macau, Macao, China; 3 Faculty of Teacher Education, Pingdingshan University, Pingdingshan 467000, China

**Keywords:** addiction-like eating, AEBS, mukbang, late-night meals, delivery food

## Abstract

Objectives: The rapid increase in Chinese obesity rates has aroused research interest in addiction-like eating (AE); however, the unavailability of assessment tools is a major barrier to further investigation. To address the research gap, this study aimed to evaluate the psychometric properties of the Addiction-like Eating Behavior Scale (AEBS) and explore associations between AE and three modern eating-related habits (i.e., ordering delivery food, eating late-night meals and watching mukbang) among Chinese adults. Methods: The STROBE checklist was followed for reporting this cross-sectional study. We conducted a telephone survey with a two-stage cluster random sampling method and acquired a probability sample of 1010 community-dwelling Chinese adults (55.2% female; age: 18–88 years, *M* = 38.52, *SD* = 14.53). Results: Results confirmed the conceptualized two-factor structure of AEBS with satisfactory model fit, reliability and validity. Chinese adults reported higher levels of AE in the context of low dietary control rather than appetitive drive. These two AE factors showed significant and positive associations with modern eating-related habits. Conclusions: The current study was the first to assess AE among Chinese adults and provided a reliable and valid assessment tool. Our correlational findings can also be utilized for designing interventions for weight management and healthy eating.

## 1. Introduction

The prevalence of obesity has increased to 13% over the past few decades worldwide, nearly tripling from 1975 to 2015 [1]. As one of the major contributing factors to this obesity surge, addictive behaviors related to food and eating have attracted growing clinical and research attention and inspired different approaches to understanding this subject [2,3]. The addictive nature of particular foods (e.g., sweet and fatty foods) [4] has been a predominate focus of study, establishing links between addictive patterns of food consumption and greater risk for being overweight and psychologically distressed [5]. Nevertheless, this approach has been criticized for its over-reliance on the substance-related addiction model and its neglect of differences between food and psychoactive substances [6,7]. Addiction-like eating, on the other hand, centers on maladaptive eating behaviors rather than food and provides a unique perspective to complement what might have been neglected by the food addiction approach [8]. Thus, addiction-like eating is an emerging approach based on the behavioral addiction model. By adopting this approach, the present study aimed to take the first step in assessing addiction-like eating in Chinese adults and explore its associations with modern eating-related habits to delineate a more comprehensive picture of addictive behaviors related to food and eating in contemporary society.

With rapid urbanization and economic development in China, problematic eating and food consumption have emerged among residents over the past few years. A report on Chinese residents’ chronic diseases and nutrition [9] showed that more than 50% of Chinese adults were obese or overweight in 2019––a rate that is expected to rise to 65.3% in 2030 [10]. Research on the food addiction approach, which frequently used the Yale Food Addiction Scale (YFAS) [4] or its later revision the modified Yale Food Addiction Scale 2.0 (mYFAS 2.0) [11], has also been frequently applied in extant Chinese studies. These studies underscored the adverse effect of food addiction on Chinese adults’ management of weight and health [12,13,14,15]; however, they also omitted the alternative approach of addiction-like eating, which could have broadened the horizon on this matter with both theoretical and practical significance [6,8,16]. One of the plausible reasons for the scarcity of studies on addiction-like eating might lie in the lack of assessment tools in the Chinese context; hence, the primary objective of the current study was to bridge this gap by examining the psychometric properties of the Addiction-like Eating Behavior Scale (AEBS) [8] among Chinese adults.

The AEBS assesses observable behaviors of addiction-like eating from the behavioral addiction perspective [8]. It was developed based on Wiers et al.’s [17] dual-process theory of motivation, which considers addiction to be an imbalance between two systems: an impulsive system, whereby pleasure is pursued through immediate gratification or reward and a reflective system that focuses on the prospect of obtaining pleasure through making use of higher order decision-making structures of the brain. This theory has been widely used to explain the development and maintenance of other behavioral addictions, such as disordered gambling [18]. In line with this theory, Ruddock et al.’s [8] AEBS consists of two components: appetitive drive (i.e., increased responsivity to reward-related cues) and low dietary control (i.e., diminished ability to exert inhibitory control over these responses). This conceptualized two-factor structure of AEBS, along with its satisfactory internal consistency and convergent validity, was found useful, not only among community-dwelling adults in the UK [8], but also in adults from Canada [19], Brazil [20] and Turkey [21]. Given the psychometric soundness of AEBS, we expected this scale to provide a reliable and valid assessment of Chinese adults’ addiction-like eating in the present study.

In addition to the elevated risk for problematic eating and food consumption, societal development in China has resulted in changes to its residents’ eating-related habits. Recent research has suggested that eating late-night meals, ordering delivery food (e.g., usually through digital applications [22]), and watching mukbang (i.e., a live-streaming host eating, often a large quantity of food, while socializing with audiences [23]) are rapidly gaining popularity among Asians [24], including Chinese people [25,26,27,28]. Although these modern eating-related habits are associated with obesity or unhealthy eating patterns [25,26,29], no research to date has examined these phenomena through the addiction-like eating approach. To address this missing link, the second objective of the present study was to explore the associations between addiction-like eating and these three modern eating-related habits (i.e., eating late at night, ordering delivery food and watching mukbang).

In sum, the present study was the first to utilize the addiction-like eating approach to better understand addictive behaviors related to food and eating in the Chinese context with two objectives; the first was to provide a valid, reliable assessment tool to evaluate addiction-like eating among Chinese adults to shed light on new perspectives on weight management and healthy eating. Specifically, our aim was to explore whether our data could support the conceptualized two-factor model of AEBS with adequate internal consistency as discovered in previous studies [19,20,21]. For indicators of convergent validity, we selected food addiction, marked distress regarding binge eating and perceived weight status, based on their consistent, positive associations with either addiction-like eating or addictive behaviors related to food and eating in general [8,30,31]. To achieve the second objective, we strived to investigate the potential impact of modern eating-related habits on addiction-like eating and anticipated discovering positive correlations between the two as indicated by extant studies [25,26,29]. We envisaged that the findings could further our knowledge of the implications of modern eating habits and inform the design of timely, cost-effective preventive strategies for unhealthy eating.

## 2. Materials and Methods

A cross-sectional telephone survey was conducted to evaluate the psychometric properties of the Chinese version of Addiction-like Eating Behavior Scale (AEBS). The measures in this survey assessed either current or recent (i.e., past 12 months) eating-related psychological or behavioral experiences of our participants. The current study followed the Strengthening the Reporting of Observational Studies in Epidemiology (STROBE) checklist for reporting cross-sectional studies [32] (see Supplementary Materials).

### 2.1. Participants and Procedures

From late 2021 to early 2022, we conducted a telephone survey using a two-stage cluster random sampling method and obtained a probability sample of 1010 community-dwelling Chinese adults (55.2% female; age: 18–88 years, *M* = 38.52, *SD* = 14.53) in Macao, China. Participants were considered eligible if they were local Chinese residents aged 18 years or above with adequate language proficiency to understand and speak Mandarin or Cantonese Chinese. Only those who gave informed consent to voluntarily participate without monetary incentives received our survey, which was anonymous and conducted by trained research assistants. According to the recommendation of an ideal size-to-parameter ratio of 20:1 in structure equation models [33], the sample size of our study was sufficient to conduct a confirmatory factor analysis. No potential sources of bias were needed to address regarding sampling. According to the calculation formula proposed by the American Association for Public Opinion Research [34], the response rate was 85.5%, which represents the proportion of the respondents who completed the survey out of the total respondents with known eligibility who were contacted. Prior ethics approval for this study was acquired from the corresponding author’s affiliated university.

### 2.2. Measures

#### 2.2.1. Addiction-like Eating

The 15-item, two-factor AEBS [8] was used to capture respondents’ addiction-like eating in the past 12 months on a 5-point Likert scale, ranging from 1 = *never* to 5 = *always*. The original English items were first translated into Chinese by two bilingual psychologists according to standardized forward–backward translation procedures [35] and with further consultation with a professional nutritionist. Additional item refinements were performed based on the pilot testing results (*n* = 5; 60% female; aged 23 to 52 years) to improve comprehensibility in the Chinese context. For example, we modified “I don’t eat a lot of high fat/sugar foods” into “I eat a lot of high fat/sugar foods” to avoid the potentially confusing double-negation associated with the frequency response of 1 = *never* to 5 = *always* (see Appendix A for the final Chinese version of AEBS). A composite sum score was computed for AEBS total to compare the overall level of addiction-like eating in the present study with those reported in previous studies. The composite mean scores of the two AEBS components were further calculated to evaluate the risks for appetitive drive and low dietary control among Chinese adults. A higher composite score represented a higher level of addiction-like eating.

#### 2.2.2. Food Addiction

We used Zhang et al.’s [15] Chinese version of the modified YFAS 2.0 (mYFAS 2.0) [11], which contains 11 items reflecting one’s food addiction symptoms. The scale was used to assess respondents’ food-related addictive symptoms (e.g., “Eating the same amount of food did not give me as much enjoyment as it used to.”); respondents rated their agreement with items on an 8-point Likert scale, ranging from 0 = *never* to 7 = *every day* during the past 12 months. The Cronbach’s alpha of the mYFAS 2.0 symptom items was 0.87 in the present study. When scoring, each item was first recoded into a binary format with the scoring key designed by Schulte and Gearhardt [11] to inform whether each symptom met the corresponding threshold; subsequently, a total score, which represents one’s symptom counts from 0 to 11, was computed by summing up how many food addiction symptoms an individual presented. Higher total scores indicated a higher level of food addiction.

#### 2.2.3. Perceived Weight Status

Similar to previous studies on Chinese adults [36], we used a single item (i.e., “How do you perceive your current weight?”) to assess respondents’ perceived weight status on a 5-point Likert scale, ranging from 1 = *underweight* to 5 = *overweight*, with a mid-point of 3 = *about the right weight*.

#### 2.2.4. Marked Distress Regarding Binge Eating

Marked distress regarding binge eating was assessed by a single item (i.e., “Did you feel very upset about your uncontrollable overeating or resulting weight gain?”) from the eating disorder diagnostic scale (EDDS) [37]. We used the Chinese version derived from Jackson and Chen’s [38] study. Participants were asked to report their levels of distress over their uncontrollable overeating or resulting weight gain in the past 12 months, on a 7-point Likert scale, ranging from 0 = *not at all* to 6 = *extremely*, which was subsequently recorded into 0 = *no* (i.e., those who scored 0 = not at all and those who reported not having uncontrollable overeating (not applicable)) and 1 = *yes* (i.e., those who scored greater than 0 = *not at all*) to indicate the presence of marked distress regarding binge eating for data analysis.

#### 2.2.5. Modern Eating-Related Habits

The three modern eating-related habits of this study’s interest were evaluated with three researcher-developed items that were relevant to the Chinese context. Participants were asked to report the frequencies of ordering delivery food, eating late-night meals and watching mukbang, respectively, in the past 12 months on a 5-point Likert scale, in which 1 = *never* and 5 = *always*.

#### 2.2.6. Demographic Information

Participants were also asked about their sex and age in the telephone survey.

### 2.3. Statistical Analysis

After preliminary data cleaning and descriptive analysis, we first examined the factorial structure of AEBS by comparing the conceptualized two-factor model, along with its alternative one-factor model with confirmatory factor analysis (CFA), with the comparative fit index (CFI) ≥ 0.90 [39], the root mean square error of approximation (RMSEA) < 0.08 and the standardized root mean square residual (SRMR) < 0.08 [40]. Each AEBS item retained in the final model was expected to be significantly accounted for by its latent parent factor and with a standardized factor loading of ≥0.30 [41]. Once the measurement model of AEBS was confirmed, we further tested the measurement invariance of AEBS across both sexes with multigroup CFA at the configural, metric and scalar levels. At each level, sex invariance was indicated if ΔCFI ≤ 0.01 and RMSEA < 0.015 [42]. All the CFAs were conducted in Mplus 8.3 [43] utilizing the robust maximum likelihood estimation (MLR) to handle missing values without assuming multivariate normality.

The remaining analyses were performed in SPSS 26.0 [44]. AEBS’s convergent validity was examined by its associations with selected indicators through bivariate correlational analysis. Internal consistency was assessed with Cronbach’s alpha (i.e., >0.70 as acceptable) [45]. We also explored and compared the associations between AEBS and three eating-related habits with bivariate correlations and hierarchical regression analyses.

## 3. Results

### 3.1. Demographic Information

Among the 1010 participants, about half of the sample was female (55.2%), with age ranging from 18 to 88 years (*M* = 38.52, *SD* = 14.53). A total of 39 participants did not report their ages, while no cases were missing reporting sexes.

### 3.2. Factorial Structure

The CFA results indicated that our data did not fit the conceptualized two-factor AEBS model satisfactorily, χ^2^(89) = 997.13, *p* < 0.001, CFI = 0.79, RMSEA = 0.10 (90% CI [0.10, 0.11]), SRMR = 0.07. After closely examining item associations, we slightly modified the model by allowing two pairs of residuals to covary between Items 11 and 12 and between Items 6 and 13. The resultant model showed good model fit, χ^2^(87) = 388.40, *p* < 0.001, CFI = 0.93, RMSEA = 0.059 (90% CI [0.053, 0.065]), SRMR = 0.05, which was superior than its alternative one-factor model, χ^2^(90) = 1331.54, *p* < 0.001, CFI = 0.71, RMSEA = 0.117 (90% CI [0.11, 0.12]), SRMR = 0.09. A further evaluation of the AEBS items showed that all were statistically significant and had adequate standardized factor loadings (0.38 to 0.85, *p* < 0.001; see Table 1), which indicated satisfactory structure validity for the two-factor AEBS.

### 3.3. Measurement Invariance across Sex

Measurement invariance was tested with multigroup CFA by configural, metric and scalar levels. As shown in Table 2, we found acceptable model fit at all the levels (CFI = 0.906 to 0.918, RMSEAS = 0.064 to 0.066, SRMR = 0.064 to 0.067) and sufficiently small differences across models (ΔCFI = 0.010 and 0.002, ΔRMSEA = 0.001 and 0.001), suggesting that the sex invariance was held at the scalar level. Further, between-group comparisons demonstrated that male participants endorsed significantly higher levels of addictive eating with respect to the AEBS total score (*M*_males_ = 26.54, *M*_females_ = 24.92, *t*(988) = –3.02, *p* = 0.001) and lower dietary control (*M*_males_ = 13.09, *M*_females_ = 11.98, *t*(996) = –4.17, *p* < 0.001) than their female counterparts. However, appetitive drive did not vary by sex (*M*_males_ = 13.44, *M*_females_ = 13.01, *t*(998) = –1.39, *p* = 0.17).

### 3.4. Internal Consistency and Convergent Validity

The Chinese version of AEBS displayed high reliability, with AEBS total score (*α* = 0.86) and its two subscales (*α* = 0.83 for appetitive drive and *α* = 0.76 for dietary control) demonstrating good internal consistency. As for convergent validity, AEBS total score was found to be positively correlated with food addiction (*r* = 0.47, *p* < 0.001), perceived weight status (*r* = 0.34, *p* < 0.001) and marked distress regarding binge eating (*r* = 0.42, *p* < 0.001). Both subscales of AEBS (i.e., appetitive drive and low dietary control) also showed significant positive correlations with food addiction (*r* = 0.51 and 0.33, *p <* 0.001), marked distress (*r* = 0.42 and 0.31, *p <* 0.001) and perceived weight status (*r* = 0.36 and 0.25, *p* < 0.001). In addition, AEBS total score was positively correlated with the scores of its two subscales, appetitive drive (*r* = 0.90, *p* < 0.001) and low dietary control (*r* = 0.88, *p* < 0.001).

### 3.5. Addiction-like Eating and Its Associations with Modern Eating-Related Habits

Addiction-like eating among Chinese adults was reflected by the composite sum score of AEBS total (*M* = 25.65, *SD* = 7.97) and the composite mean scores of the two AEBS components (appetitive drive: *M* = 1.47, *SD* = 0.54; low dietary control: *M* = 2.08, *SD* = 0.70). The endorsement rate in Table 1 shows that 0.7% to 19.6% of Chinese adults in the present sample reported “often” or “always” engaging in various addiction-like eating behaviors during the past 12 months. When comparing the two addiction-like eating components, Chinese adults in our sample endorsed higher rates of low dietary control behaviors (3.9–19.6%) than appetitive drive behaviors (0.7–8.2%) overall. Regarding low dietary control, the two most endorsed addition-like eating behaviors were being “never” or “rarely” “easily able to make healthy food choices” (Item 6; 19.6%) and “maintaining a healthy diet” (Item 13; 14.8%). In appetitive drive, the two most highly endorsed items (always or often) were “find(ing) it difficult to limit what/how much I eat” (Item 3; 8.2%) and “feel(ing) unable to control my weight” (Item 15; 6.7%).

Among the 1010 participants, 868 participants (85.9%) reported ordering delivery food, with 692 (68.5%) reporting eating late-night meals and 407 (40.6%) having watched mukbang in the past 12 months. The results of bivariate correlations suggested that AEBS and its two subscales were significantly correlated with the three eating-related habits, with a small effect size for ordering delivery food (*r* = 0.11 to 0.26, *p* < 0.001) and watching mukbang (*r* = 0.17 to 0.19, *p* < 0.001) and a moderate effect size for eating late-night meals (*r* = 0.32 to 0.43, *p* < 0.001). As shown in Table 3, when these three eating-related habits were being controlled for simultaneously, both eating late-night meals and watching mukbang (*p <* 0.001), but not ordering delivery food (*p >* 0.05), were significantly associated with AEBS total score (∆*R*^2^ *=* 0.190, *p* < 0.001) and appetitive drive score (∆*R*^2^ = 0.121, *p* < 0.001); in contrast, all three eating-related habits (*p <* 0.05) were positively related to low dietary control scores (∆*R*^2^ = 0.195, *p* < 0.001).

## 4. Discussion

Responding to the growing body of literature pertaining to problematic control of weight and eating in China, the present study employed a promising but under-researched approach, addiction-like eating, to gain a better understanding of addictive behaviors related to food and eating through two objectives. Regarding our first objective, to facilitate the assessment of addiction-like eating in the Chinese context, our psychometric evaluations showed that AEBS provides a reliable and valid assessment of addiction-like eating among Chinese adults. Specifically, we replicated the conceptual two-factor structure from Ruddock et al.’s original research [8]; namely, the two components of appetitive drive and low dietary control and our data showed a satisfactory model fit and adequate standardized factor loadings. Our finding was consistent with research validating the AEBS in other countries (e.g., Canada and Turkey) [19,21] and provides additional support for the cross-cultural applicability of the addiction-like eating approach. We also found measurement invariance across sex at the scalar level for this two-factor structure, which offers the first piece of information regarding the construct consistency of AEBS between sex groups. Satisfactory internal consistency was found for AEBS as a whole and its two subscales, which is consistent with previous studies [8,19]. The good convergent validity of AEBS was evidenced by significant, positive correlations with food addiction, marked distress regarding binge eating and perceived weight status, which is in line with previous literature [8,30,31].

When applying the AEBS to evaluate overall addiction-like eating levels in the Chinese context, we found that Chinese adults might be at a relatively lower risk for addiction-like eating (*M* = 25.65, *SD* = 7.97) than adults in the UK (*M* = 41.41, *SD* = 9.83) [8], Canada (*M* = 36.35, *SD* = 10.25) [19], Turkey (*M* = 40.43, *SD* = 8.35) [21] and Brazil (*M* = 41.19, *SD* = 10.93) [20]. Nevertheless, our finding regarding higher levels of low dietary control than appetitive drive was consistently observed across all studies, suggesting that more attention must be paid to dietary interventions. In parallel to this finding, our results of endorsement rates also revealed that having difficulties maintaining a healthy diet and making healthy food choices were the two most frequent addiction-like eating symptoms reported by Chinese adults. In fact, lifestyle dietary interventions, such as reduced-calorie diets, are already considered to be first-line interventions for obesity according to the latest Chinese nutrition experts’ consensus, albeit that more empirical evidence is needed to examine the long-term effectiveness of these types of interventions [46]. Taken together, we recommend that future studies empirically evaluate the effects of lifestyle dietary interventions for preventing addictive behaviors related to food and eating, as well as for weight problems in the long run.

Drawing from the measurement invariance of AEBS across sex, we further discovered that male participants reported significantly higher levels of addiction-like eating as a whole and lower dietary control than female participants. Although previous studies have not reported significant sex differences in addiction-like eating, we could not fully rule out the possibility that the nonsignificant sex difference in the UK and Brazil was partly due to a disproportionate sex composition in the sample (i.e., 86% female) [8] or relatively small sample size (*N* = 150) [20]. Thus, we call for more research to be conducted with sufficiently large samples that reflect equal sex representation in order to further examine sex differences in addiction-like eating cross-culturally for its potential application on sex-specific intervention designs.

Regarding our second objective, as expected, we uncovered significant, positive associations (via bivariate correlations) between modern eating-related habits and addiction-like eating, which extends previous research findings that show similar associations between these eating-related habits and obesity or unhealthy eating patterns in general [25,26,27]. Of the three modern eating-related habits, eating late-night meals appeared to exert the strongest influence on addiction-like eating (i.e., moderate correlation), whereas the other two habits of ordering delivery food and watching mukbang (i.e., mild correlation) exerted a milder influence. Considering the high percentage (i.e., 40.6 to 85.9%) of our respondents who reported engaging in these habits during the past 12 months, we recommend psychoeducational campaigns among the public on the heightened risk for addiction-like eating due to these eating-related habits, especially with respect to eating late-night meals. As these habits are consistently associated with overweight issues [47,48], regular assessment of addiction-like eating is also warranted for those who practice these habits frequently; such assessments might have a tempering effect on the growing population suffering from obesity or being overweight in China.

When the three eating-related habits were taken into account simultaneously in regressions, we consistently observed that the most salient impact on addiction-like eating as a whole, as well as on low dietary control and appetitive drive, was from eating late-night meals, followed by watching mukbang, In contrast, the impact of ordering delivery food was found to have significance only on low dietary control, but not on addiction-like eating as a whole or appetitive drive, after controlling for the other two habits. Taken together, if the resources for interventions are limited, we advise stakeholders to prioritize targeting the habits of eating late-night meals and watching mukbang. Given their recent increasing popularity [49], especially among young populations of adolescents [50] and university students [51], smartphone applications related to food could be utilized as a platform to provide easily accessible information on dietary control and healthy food choices to those who order late-night meals and watch mukbang through smartphone applications.

Our study has several limitations worth noting. First, the cross-sectional design used in the present study could not assess the temporal variations of addiction-like eating among Chinese adults, nor provide additional information on AEBS’s test-retest reliability or predictive validity. We recommend that future researchers adopt a longitudinal research design to address these limitations. Second, although our probability sample is able to render sufficient power to make generalizations, a telephone survey design may lower the generalizability due to its disadvantages including physical/language requirements (e.g., normal hearing), limited calling time (i.e., 6 pm to 10 pm on weekdays, 2 pm to 10 pm on weekends in this study) and available telephone numbers (e.g., based on Macao residential phonebook in this study). Additionally, our findings can only be used to understand the general community-dwelling Chinese adults, not younger individuals or clinically obese populations. Future studies are encouraged to extend the assessment of addiction-like eating to more diversified populations in the Chinese context in order to establish a norm for AEBS and identify its optimal cutoff point for identifying those who are prone to addiction-like eating. Third, we only explored three types of modern eating-related habits in the current study as an initial attempt. Subsequent research may include other emerging modern eating-related habits to facilitate early detection of other risk factors associated with addiction-like eating in modern times.

## 5. Conclusions

Despite the limitations, our study provided the first piece of empirical evidence on addiction-like eating in the Chinese context as a way to mitigate the obesity surge and promote healthy eating. Based on a probability adult sample, we not only offered a reliable and valid assessment tool, the Chinese version of AEBS, to evaluate addiction-like eating among Chinese adults from the two aspects of low dietary control and appetitive drive but also laid the empirical foundation of the dual-process model for subsequent research, to further study addictive behaviors related to food and eating with the addiction-like eating approach in China. Moreover, our findings also highlighted the correlations between modern eating-related habits and addiction-like eating and further investigation on the biopsychosocial mechanism(s) underlying such correlations must be conducted for a more holistic understanding of (un)healthy eating patterns.

Our findings on addiction-like eating among Chinese adults also provide some practical implications. For instance, the significant relationships between modern eating-related habits and addiction-like eating indicated emerging risks for the adversity associated with food and eating in the contemporary world and shed light on new directions for early identification and intervention of problematic eating. Given the potential of researching addiction-like eating to inform designs targeting problems associated with food and eating, we recommend further application of AEBS to track addiction-like eating in the Chinese context and evaluate its risks and associated consequences over time. Furthermore, we advocate for the design of tailored psychoeducational programs for individuals who regularly engage in these problematic modern eating-related habits and for stakeholders who provide food-related service (e.g., smartphone-based delivery food applications) in order to lower the risks of individuals developing addictive behaviors related to food and eating, as well as weight problems among the general public. We further recommend the promotion of lifestyle dietary interventions for at-risk populations with low dietary control.

## Figures and Tables

**Table 1:** Standardized factor loading and item endorsement of AEBS (*N* = 1010).

Factors/Items	Factor Loading ^a^	Endorsement Rate ^c^
**Factor 1: Appetitive drive**		
1. I continued to eat despite feeling full	0.52	1.9%
2. I serve myself overly large portions	0.78	1.7%
3. I find it difficult to limit what/how much I eat	0.44	8.2%
4. I binge eat	0.80	0.8%
5. When it comes to food, I tend to over-indulge	0.80	3.2%
7. Once I start eating certain foods, I can’t stop until there’s nothing left	0.50	5.4%
9. I eat until I feel sick	0.70	0.7%
14. I tend to overeat	0.85	2.3%
15. I feel unable to control my weight	0.47	6.7%
**Factor 2: Low dietary control**		
6. I am easily able to make healthy food choices (R)	0.38 ^b^	19.6% ^d^
8. Despite trying to eat healthy, I end up eating ‘naughty’ foods	0.56	8.4%
10. I continue to eat certain unhealthy foods despite being aware of their effects on my health	0.73	6.6%
11. I buy processed foods that are high in fat and/or sugar	0.55	4.0%
12. I eat a lot of high fat or sugar foods	0.65	3.9%
13. I maintain a healthy diet (R)	0.45 ^b^	14.8% ^d^

Note. (R) = Reverse scored. ^a^ All the AEBS items displayed a significant level of < 0.001. ^b^ Denotes items being reverse-scored before the confirmatory factor analysis. ^c^ The proportion of those who rated *often* or *always* on the corresponding item unless specified otherwise. ^d^ Denotes the proportion of those who originally rated *never* or *rarely* on the two reversed items before being reverse-scored.

**Table 2:** Measurement invariance of AEBS across both sexes (*N* = 1010).

Model	Model Fit						Model Comparison				
	χ^2^	*df*	*p*	CFI	RMSEA [90% CI]	SRMR	Δχ^2^	Δ*df*	*p*	ΔCFI	ΔRMSEA
Configural	552.01	174	<0.001	0.918	0.066 [0.059, 0.072]	0.067					
Metric	572.62	187	<0.001	0.916	0.064 [0.058, 0.070]	0.064	28.02	13	0.009	0.002	0.001
Scalar	632.24	200	<0.001	0.906	0.065 [0.060, 0.071]	0.067	61.14	13	<0.001	0.010	0.001

**Table 3:** Hierarchical regressions exploring relationships between three eating-related habits and AEBS.

	AEBS Total (*N* = 983)				AEBS Appetitive Drive (*N* = 993)				AEBS Low Dietary Control (*N* = 991)			
	*β*	*B*	[95% CI]	*t*	*β*	*B*	[95% CI]	*t*	*β*	*B*	[95% CI]	*t*
**Step 1**												
(Constant)		24.86	[24.19, 25.53]	73.03 ^***^		13.00	[12.59, 13.40]	63.18 ^***^		11.95	[11.60, 12.30]	66.68 ^***^
Sex	0.11	1.70	[0.71, 2.70]	3.36 ^**^	0.05	0.45	[−0.15, 1.06]	1.48	0.14	1.16	[0.64, 1.68]	4.34 ^***^
	∆*R*^2^ = 0.011, *p* = 0.001				∆*R*^2^ = 0.002, *p* = 0.139				∆*R*^2^ = 0.019, *p* < 0.001			
**Step 2**												
(Constant)		15.62	[14.07, 17.17]	19.82 ^***^		8.87	[7.90, 9.84]	17.90 ^***^		6.68	[5.87, 7.49]	16.21 ^***^
Sex	0.05	0.83	[−0.12, 1.77]	1.72	0.01	0.13	[−0.46, 0.72]	0.42	0.07	0.63	[0.14, 1.12]	2.51 ^*^
Order delivery food	0.04	0.32	[−0.14, 0.77]	1.38	−0.02	−0.11	[−0.39, 0.18]	−0.73	0.10	0.38	[0.15, 0.62]	3.19 ^**^
Eat late-night meals	0.37	3.33	[2.77, 3.90]	11.63 ^***^	0.31	1.69	[1.34, 2.04]	9.38 ^***^	0.36	1.72	[1.43, 2.01]	11.54 ^***^
Watch mukbang	0.16	1.14	[0.71, 1.57]	5.23 ^***^	0.15	0.67	[0.40, 0.93]	4.89 ^***^	0.14	0.53	[0.31, 0.75]	4.66 ^***^
	∆*R*^2^ = 0.185, *p* < 0.001				∆*R*^2^ = 0.121, *p* < 0.001				∆*R*^2^ = 0.195, *p* < 0.001			

Note. ^*^ *p* < 0.05, ^**^ *p* < 0.01, ^***^ *p* < 0.001.

## Data Availability

The data presented in this study are available on request from the corresponding author.

## Supplementary Materials

The following supporting information can be downloaded at: https://www.mdpi.com/article/10.3390/nu14224836/s1, Table S1: STROBE Statement—checklists of items that should be included in reports of observational studies.

## Appendix A

**[**指导语**]下面是一些关于饮食行为及习惯的描述, 请问在过去的 12 个月中, 你有多经常经历以下的情況? 请用 1–5 评分, 1 = 从不, 2 = 很少, 3 = 有时, 4 = 经常, 5 = 总是. [Instructions] The following statements describe eating behaviors and habits. How often have you experienced the following situations in the past 12 months? 1 = Never, 2 = Rarely, 3 = Sometimes, 4 = Most of the time, 5 = Always.**

**Table A1:** The Chinese version of Addiction-like Eating Behavior Scale (类成瘾性饮食行为量表中文版).

	从不 (Never)	很少 (Rarely)	有时 (Sometimes)	经常 (Most of the time)	总是 (Always)
1. 就算感觉吃饱了, 我也会继续吃 (I continued to eat despite feeling full)	1	2	3	4	5
2. 吃东西的时候, 我给自己准备超大份的食物 (I serve myself overly large portions)	1	2	3	4	5
3. 我感到很难限制自己吃什么或吃多少 (I find it difficult to limit what/how much I eat)	1	2	3	4	5
4. 我有暴饮暴食 (I binge eat)	1	2	3	4	5
5. 在饮食上, 我过度放纵自己 (When it comes to food, I tend to over-indulge)	1	2	3	4	5
6 *. 对我来说, 选择健康饮食是件容易做到的事情 (I am easily able to make healthy food choices)	1	2	3	4	5
7. 我一旦开始吃有些食物, 就必须要全部吃完才能停下来 (Once I start eating certain foods, I can’t stop until there’s nothing left)	1	2	3	4	5
8. 尽管我尝试想要吃得更健康, 我最终还是吃了不健康的食物 (Despite trying to eat healthy, I end up eating ‘naughty’ food)	1	2	3	4	5
9. 我会吃到自己难受才停下来 (I eat until I feel sick)	1	2	3	4	5
10. 尽管我意识到某些食物影响了我的健康, 我还是继续吃这些不健康的食物 (I continue to eat certain unhealthy foods despite being aware of their effects on my health)	1	2	3	4	5
11. 我有买脂肪及(或)糖份含量高的加工食品 (I buy processed foods that are high in fat and/or sugar)	1	2	3	4	5
12. 我有大量进食高脂,高糖的食物 (I eat a lot of high fat or sugar foods)	1	2	3	4	5
13 *. 我有保持健康的饮食习惯 (I maintain a healthy diet)	1	2	3	4	5
14. 我有过度进食(吃得过饱) (I tend to overeat)	1	2	3	4	5
15. 我感到无法控制自己的体重 (I feel unable to control my weight)	1	2	3	4	5

备注: (1) 条目1, 2, 3, 4, 5, 7, 9, 14和15为食欲驱动因子, 条目 6, 8, 10, 11, 12和13为低饮食控制因子. (2) * 标记的条目6及13为反向计分. *Note:* (1) Appetitive drive: Items 1, 2, 3, 4, 5, 7, 9, 14 and 15; Low dietary control: Items 6, 8, 10, 11, 12 and 13. (2) * Item 6 and Item 13 are reverse-scored.
